# Polymer Coating of Carbon Nanotube Fibers for Electric Microcables

**DOI:** 10.3390/nano4040879

**Published:** 2014-11-04

**Authors:** Noe T. Alvarez, Timothy Ochmann, Nicholas Kienzle, Brad Ruff, Mark R. Haase, Tracy Hopkins, Sarah Pixley, David Mast, Mark J. Schulz, Vesselin Shanov

**Affiliations:** 1 Nanoworld Laboratories, University of Cincinnati, Cincinnati, OH 45221, USA; 2 Chemical and Materials Engineering, University of Cincinnati, Cincinnati, OH 45221, USA; E-Mails: ochmantw@mail.uc.edu (T.O.); kienzlnn@mail.uc.edu (N.K.); haasemr@mail.uc.edu (M.R.H.); 3 Mechanical Engineering and School of Dynamics, University of Cincinnati, Cincinnati, OH 45221, USA; E-Mails: brad.m.ruff@gmail.com (B.R.); schulzmk@ucmail.uc.edu (M.J.S.); 4 College of Medicine, University of Cincinnati, Cincinnati, OH 45221, USA; E-Mails: morttl@ucmail.uc.edu (T.H.); sarah.pixley@uc.edu (S.P.); 5 Physics Department, University of Cincinnati, Cincinnati, OH 45221, USA; E-Mail: david.mast@uc.edu

**Keywords:** carbon nanotubes (CNTs), coating, doping, microcable, densification

## Abstract

Carbon nanotubes (CNTs) are considered the most promising candidates to replace Cu and Al in a large number of electrical, mechanical and thermal applications. Although most CNT industrial applications require macro and micro size CNT fiber assemblies, several techniques to make conducting CNT fibers, threads, yarns and ropes have been reported to this day, and improvement of their electrical and mechanical conductivity continues. Some electrical applications of these CNT conducting fibers require an insulating layer for electrical insulation and protection against mechanical tearing. Ideally, a flexible insulator such as hydrogenated nitrile butadiene rubber (HNBR) on the CNT fiber can allow fabrication of CNT coils that can be assembled into lightweight, corrosion resistant electrical motors and transformers. HNBR is a largely used commercial polymer that unlike other cable-coating polymers such as polyvinyl chloride (PVC), it provides unique continuous and uniform coating on the CNT fibers. The polymer coated/insulated CNT fibers have a 26.54 μm average diameter—which is approximately four times the diameter of a red blood cell—is produced by a simple dip-coating process. Our results confirm that HNBR in solution creates a few microns uniform insulation and mechanical protection over a CNT fiber that is used as the electrically conducting core.

## 1. Introduction

Carbon nanotubes (CNTs) are promising materials for the development of new technologies due to their unique physical properties, which include good mechanical strength [1,2,3], heat transport [4,5] and electrical conductivity [6,7]; many of these properties surpass those seen in other materials. Assembling CNT fibers into useful materials has proven to be challenging, and has thus far limited the use of these nanomaterials. Researchers have been devising unique methods of assembling individual or bundled CNTs into macroscopic fibers [8,9,10,11,12,13], including: gas phase spinning [10], liquid phase spinning [11,14], dry spinning [8,15,16], electrospinning [17], and variations on these techniques [18,19]. Each technique has unique advantages and disadvantages; however, none of these techniques has yet produced fibers or yarns with the same properties as individual CNTs. It has been suggested that using CNT starting materials with longer CNTs and fewer impurities would allow for the fabrication of CNT yarns with mechanical and electrical properties that are more representative of the properties of individual CNTs [20].

**Figure 1:**
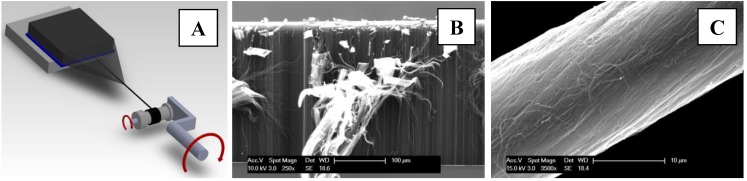
Diagram and images that illustrate: (**A**) the dry spinning process of carbon nanotubes (CNTs) fibers/threads from spinnable vertical arrays of CNT forest; (**B**) the scanning electron microscopy (SEM) of vertical array of CNT forest that is used as the starting materialfor making CNT fibers; and (**C**) the SEM image of typical CNT fiber used in our experiments.

One commonly used approach of CNT fiber mass manufacturing process is the gas phase fiber spinning, initially reported by Zhu *et al.* [9] and Motta *et al.* [21] It can produce CNT fibers without interruption. Unfortunately, the catalyst nanoparticles generated during this process act as a contaminant, reducing fiber purity, disrupting the structure, and lowering the tensile properties of the as-spun material [20,22]. Wet spinning CNT fibers follow a more conventional process similar to ones used in polymer fibers. CNTs are dispersed in solvents, sometimes using additives, and others are dispersed in acids. CNTs dispersed in strong acids were used to spin highly conducting CNT fibers [11,14,23,24]. Dry spinning from spinnable vertically aligned (VA) CNT forests is also popular, due to its facile approach of fiber spinning directly from as-grown CNT arrays [8,15,25,26]. Dry spinning from forests, first reported by Jiang *et al.* [8], allows the preparation of CNT fibers with aligned CNT bundles, and reduces metal nanoparticle contaminants within the yarn. Figure 1A illustrates the dry spinning method from VA CNT arrays that are assembled into a yarn by pulling and twisting a uniform ribbon of aligned CNT bundles from the as-grown CNT array.

Spinnable CNT yarns have become important in developing new applications since their first discovery in 2002 [8]. These yarns have allowed the fabrication of diverse commercial products, such as touch screens, liquid crystal displays (LCD), transparent loudspeakers and transmission electron microscopy (TEM) nanogrids [27,28]. Other potential products, such as electromagnetic interference (EMI) shielding films, composites, and sensors are at different stages of production and commercialization [27,28,29,30,31]. Yet, there are many applications under study, especially within electronic devices and sensors, the CNT threads, yarns and fibers, terms used indistinctly within the literatures [28,32,33], wherein an electrical conductive fiber must have an insulating coating to be of use. Because the hydrophobic nature of CNTs and the small diameter of the fibers, typically between 20 µm and 30 µm, most of the polymers used for coating purposes do not have affinity for CNTs.

In this manuscript, we report the uniform polymer coating of CNT fibers by a simple dip-coating approach. The process consists of dipping the CNT fiber into a polymer solution, and pulling out the polymer coated yarn at a constant speed. The polymer coating serves to electrically insulate the fiber, which can then be used as a conducting component in many devices. Additionally, the coating also protects CNT threads from mechanical tearing upon contact. The success of this approach to electrical insulation is demonstrated by manufacturing a two-ply conducting microcable, which is used in a simple circuit with a battery powered light-emitting diode (LED). This coating approach is simple, scalable, and allows us to precisely modify the thickness of the coating.

## 2. Materials and Methods

A typical procedure for our polymer coated CNT fibers includes synthesis of VA spinnable CNTs, spinning the arrays into threads/fibers, densification using organic solvents, doping, and then polymer coating, followed by polymer curing. The multi-walled CNTs used for this work were synthesized in accordance with procedures previously reported by Jayasinghe *et al.* [25]. The as-synthesized CNT arrays were spun into yarns by drawing a CNT web from the array, while simultaneously twisting that web, as shown in Figure 1A; a typical spinnable CNT array shown in Figure 1B, has ~300 μm tall CNTs within the array. The as-spun CNT fiber is shown in Figure 1C.

The polymer used in this study was Zetpol 2000 hydrogenated nitrile butadiene rubber (HNBR) from Zeon Chemicals L. P. (Louisville, KY, USA). According to the manufacturer, this polymer has 49% acrylonitrile (ACN) content and Mooney viscosity of 65 ML(1 + 4) at 100 °C. To prepare the coating solution, 10 g of HNBR were dispersed in 450 mL of acetone (Sigma Aldrich, St. Luis, MO, USA), without any additional treatment. After complete dissolution of the HNBR in acetone, the mixture was concentrated by solvent evaporation, to increase its viscosity, by permanently stirring until the volume of the polymer solution was reduced to 180 mL. For polymer curing, a total of 0.5 g of 2-5-bistertbutylperoxy 2-5-dimethyl hexane was then added dropwise, and stirred for 5 min.

The as-spun CNT thread was dipped into the prepared polymer solution. The polymer coated fiber was drawn from the solution at ~9 rpm constant speed, onto an 8 mm diameter bobbin. A miniature furnace, in-house built and operating at 160 °C, was placed between the collecting bobbin and the delivering bobbin, so that the freshly polymer-coated CNT fiber passes through the center of the furnace for polymer curing. Prior to coating, some yarns were immersed in an Au salt solution, in order to promote exo-hedral doping of the CNTs. The solution used for dipping was an aqueous KAuCl_4_ (0.05 M) (Sigma Aldrich), prepared in accordance with the procedures described by Jarosz *et al.* [34]. Resistivity measurements were performed at atmospheric pressure and temperatures, on a custom 4-probe testing apparatus that uses a modified commercial 5-pin connector, as shown in the inset.

**Figure 2:**
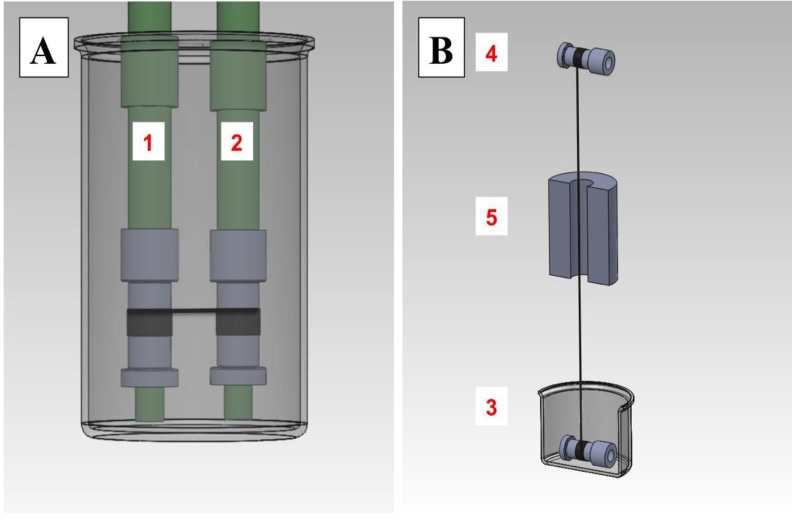
Diagrams of CNT-thread densifying and polymer-coating apparatus: (**A**) densifying apparatus with bobbin (1) and bobbin (2), as the delivering and collecting spools, respectively, immersed within the densifying solvent; (**B**) polymer coating apparatus with delivering spool (3) and the collecting spool (4) aligned vertically. Miniature furnace (5) is located within the collecting and delivering spools for the polymer curing.

Custom densifying and polymer coating setups were constructed as shown in Figure 2. The densifying machine, shown in Figure 2A, consists of a gear box connected to two shafts; these shafts serve as bobbin mounts. The gearing can be varied to cause the collecting bobbin to rotate 1%–5% faster than the delivering bobbin. The polymer coating apparatus, shown in Figure 2B, consists of a vessel, in which the delivering bobbin is immersed in the polymer solution; the bobbin is supported by a rod, and is free to rotate. The speed of the collecting bobbin is controlled by a DC electrical motor, which applies tension while pulling the polymer coated CNT thread. The miniature furnace, described in Figure 2B, is a simple tube furnace, with resistive heating elements. The temperature of the mini-furnace is maintained using a thermocouple and microcontroller as a temperature-control feedback loop.

Scanning electron microscopy (SEM) micrographs were collected using an environmental scanning electron microscopy ESEM Philips XL-30 Field Emission (Hillsboro, OR, USA). Raman spectra were collected using Renishaw In Via micro Raman Spectrometer (Hoffman States, IL, USA). Electrical conductivity of the fibers was measured with a home-made 4-point probe setup that has four electrodes in series where current pass through the outer electrodes and voltage is measured within the inner electrodes. This setup allowed us to get reproducible measurements of the fibers as wells as metallic thin wires.

## 3. Results and Discussion

Densification—also known as solvent shrinking [33]—is a simple processing step with the purpose of increasing the density of the CNT fiber, simultaneously increasing contact area between the CNTs in the fiber. This can be done using organic solvents such as acetone, alcohols [1,16,33], as well as by applying mechanical pressure [35]. The consensus is that CNTs in as-spun fibers have large inter-tube spacing; despite a compact cylindrical appearance, the yarn diameter tends to shrink when immersed into organic solvents. By reducing the spacing between CNTs, thereby increasing the Van der Waals attraction between them, densification increases the tensile strength of a CNT yarn; however, it also reduces the yarn’s yield strain. Additionally, densification typically increases the conductivity of the yarn. Depending on the affinity of the solvent for CNTs and its surface tension, different diameter reductions have been reported. Liu *et al.* [33] has suggested acetone as the ideal solvent. Reported modifications of the as-spun yarn density increase the contact between the CNTs, although the opposite effect is also possible. In order to perform more systematic densification, a fixture shown in Figure 2A was constructed, where the collecting bobbin normally spins 1% faster than the delivering bobbin, in order to provide additional tension during densification.

CNT fibers can be considered a promising material for the future of electron transport, due to their high electrical conductivity, resistance to corrosion, low weight, and high flexibility and resistance to fatigue [29]. Indeed, some of these properties surpass those of Cu wire, suggesting that CNT yarns could be an excellent conducting material allowing fabrication of thinner wires with higher flexibility. Most common electrical wires and cables need an insulating coating, to protect against electrical shorting between the conducting cores in a device (e.g., wires in a coil), or between the wires and the environment. The thickness of the insulating coating depends mostly on the conductor application. Some applications require thick insulation such as seen in typical wires and cables while other small diameter Cu wires, some known as magnet or enamel wires used as coils in most electrical motors, have about 20 µm of thin insulating coating that prevents electrical contact between adjacent wires in the coil. Polymers, such as polyethylene (PE), polyvinyl chloride (PVC) and polypropylene are typically used as insulation coating in modern devices and cabling. Because most of the cable coatings are done with extrusion processes, exploring CNT coatings with extrusion as reported by Lekawa-Raus *et al.* [36], seems a simple size reduction. Unfortunately, there are some limitations of applying extrusion coating in CNT fibers, one of them is the aspect-ratio between CNTs and the coating polymer. The extrusion approach can bury the conducting core within excessive polymer coating, where polymer represents more than 2/3 of the cable diameter as shown in images by Lekawa-Raus *et al.* [36].

An effective polymer insulator for CNT yarns must meet certain criteria: it should have a good affinity for the CNT surface, typically hydrophobic; it should be flexible; it should be electrically insulating; and it should be abrasion resistant. Most of the polymers will meet these criteria; however, for dip-coating purposes the most determinant factor is the polymer affinity to the CNT. Among the polymers that are currently being explored at the Nanoworld Laboratories in University of Cincinnati (Cincinnati, OH, USA) for this purpose, HNBR is one of the most promising candidates. HNBR has been shown to effectively disperse CNTs, suggesting a good affinity for CNTs [37,38]. Also, HNBR has a good flexibility (as compared to CNT yarns), is insulating (like most polymers), and it has the additional advantage of being resistant to oils and H_2_O.

The dipping-coating principle is shown in Figure 2B, the collecting bobbin pulls the thread at a set speed by rotating on its own axis, the shaft of a DC electric motor. Increasing the speed of the collecting bobbin increases the tension of the CNT thread during coating. A pulling speed near 9 rpm (8 mm diameter bobbin) is normal, but this is varied according to the CNT fiber diameter; thinner fibers need slower speeds to avoid breakage. Increasing the diameter of the fiber, or the number of plies in the fiber can also reduce breakage by increasing the breaking force. Increasing the diameter requires wider uniform-spinnable CNT arrays, as CNT yarn diameter is directly proportional to the width of the spinnable CNT arrays. Increasing the number of plies can also increase the diameter of the CNT yarn; however, making multiple plies requires an even-twist on each conforming ply. Small twisting mismatches between the plies generate localized stress concentrations, which tend to form twists in the fiber if the tension along the yarn axis is reduced (Figure S1). These loops and twisted sections remain even after polymer coating; they can untwist suddenly with changes in tension, tearing the polymer coating. Due to the difficulties associated with CNT fibers made of multi-ply yarns, our results are solely based on single ply CNT yarns between 20 µm and 35 µm in diameter, and tens of meters long in length, which can be manipulated and processed without breaking.

**Figure 3:**
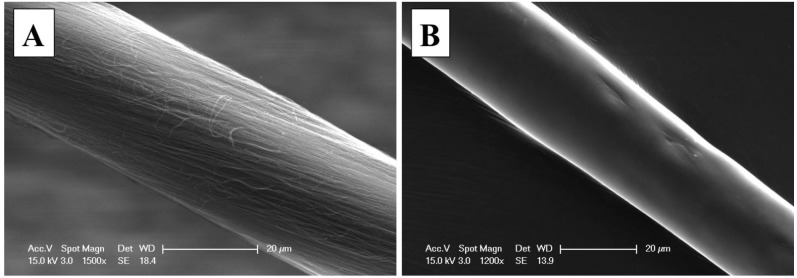
SEM images of (**A**) pristine and (**B**) polymer coated CNT fibers.

As this coating method is based on a single dip coating, HNBR concentration in the solution is an important factor to consider. It is found that an HNBR polymer concentration of about 5.5 g/100 mL leaves a uniform and continuous film along the CNT fiber. Higher polymer concentrations resulted in large polymer aggregates along the fiber, which affect the uniformity of the coating. Conversely, more dilute solutions left thinner or non-coated fiber sections. Based on the coating thickness measurements, (about 4 μm), we estimate that a typical 5 m long thread will consume less than 0.1% of polymer. Non-coated or as-spun and evenly coated CNT fiber sections are shown in Figure 3; the coated fiber (~5 m long) was prepared using near-optimum concentrations and has 26.54 μm (σ = 3.15) average diameter, compared to 27.81 μm (σ = 1.84) of the as-spun for 20 measurements along the 5 m fiber. The difference in diameter is associated to the CNT fiber densification after coating which is typical of CNT fibers upon organic solvent diffusion within the fiber; up to 24% CNT fiber diameter shrinking in acetone has been reported [33]; however, we have observed shrinking in up to 48%.

**Figure 4:**
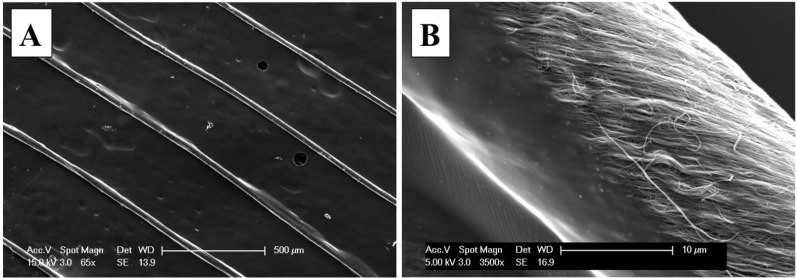
SEM images of several coated CNT fibers that: (**A**) illustrate the uniform nature of the polymer coating; and (**B**) show the interface of the CNT fiber and its coating polymer.

Figure 4A confirms polymer coating uniformity by imaging five sections of the yarn. SEM Images taken at the interface of the polymer coating and CNT yarn (Figure 4B) reveal the wetting quality of the polymer. This image was taken at the initial coating point of the CNT fiber, which corresponds to sections of the immersed and the exposed CNT fiber sections that is used to draw the fiber from the polymer solution.

**Figure 5:**
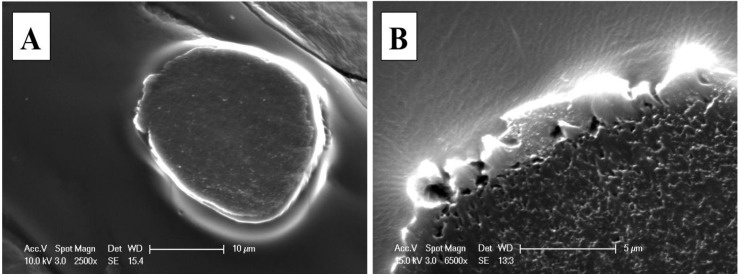
(**A**) SEM images of cross section of the polymer coated CNT thread; and (**B**) higher magnification of the cross section that illustrates the CNT packing within the CNT fiber.

Attempts to quantify the polymer coating thickness, by cutting the CNT fiber with a cryostat (a freezing microtome), had limited success. SEM images in Figure 5 illustrate the cross section of the polymer-coated CNT fiber, which was immersed in a standard H_2_O-soluble glycol and resin mixture, known as optimum cutting temperature (OCT) compound, to perform the cutting at −10 °C. The SEM image in Figure 5A suggests that the thin coating was damaged by the microtome during cutting. As shown in Figure 5B, a higher magnification image of the coated CNT yarn reveals small cracks in the coating polymer near the edge of the thread, which suggests that pressure applied by the microtome on the thin polymer coating has damaged the polymer coating. Glass transition temperature of HNBR, *T*_G_ is equal to −40 °C, is close to the cutting temperature (−10 °C) but at this temperature the polymer may be still soft enough. However, the images of microtome cuts of coated CNT fibers reveal the circular nature of their cross section, supporting the use of a cylindrical assumption in determining the electrical resistivity of the CNT fibers.

**Figure 6:**
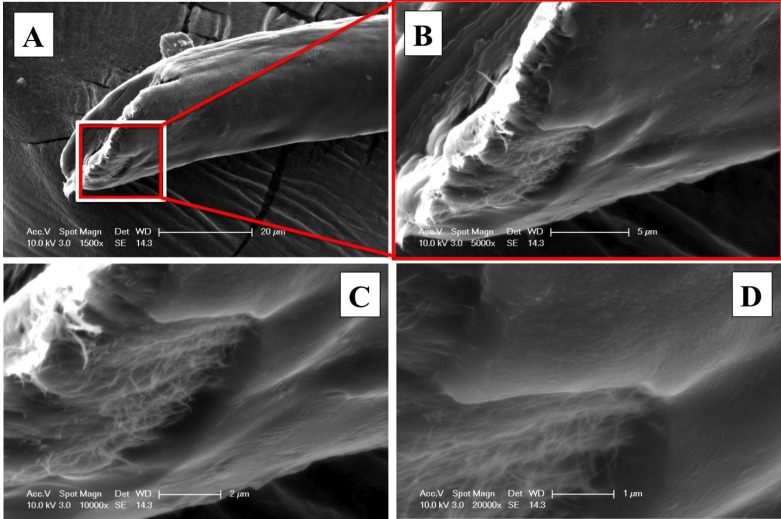
SEM micrographs of the cut-end of a polymer coated CNT fiber cut in cross section that allows the visualization of the polymer coating thickness with ever increasing magnification: (**A**) 1500×; (**B**)5000×; (**C**) 10,000×; and (**D**) 20,000×.

Additional attempts to determine the thickness of the coating have been performed. A common razor blade was used to cut the microcables at ambient temperature and then their cross-sections were examined under SEM. As expected, and as shown in Figure 6, the cross section was damaged and no longer shows a uniform circular fiber; however, a search of several fiber-cuts revealed sections where the thickness of the polymer could be evaluated. As shown in Figure 6A, the cut-end of the CNT fiber reveals some tearing where it is possible to observe the morphology and thickness of the polymer coating qualitatively. Figure 6B–D is higher magnification image, shown in increasing order, showing the polymer tearing area at the cut-end. These higher magnifications prove polymer coating was complete around the CNT fiber circumference and suggest a relatively uniform thickness. These images support the idea that the cracks observed in the cryostat-cut sample, Figure 5B, are the result of cutting at temperatures below 0 °C. Furthermore, these images reveal that despite the good polymer wetting along the CNT fiber surface as shown in Figure 4B, the polymer does not deeply penetrate within the yarn. This is also supported by the cryostat-cut cross-section, where the polymer is located around the fiber circumference only. It is important to avoid polymer penetration within the fiber, as it may negatively affect the electrical conductivity of the CNT microcable.

**Figure 7:**
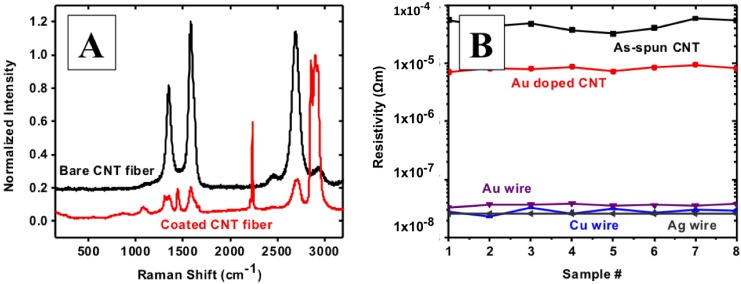
(**A**) Raman characterization of CNT fibers before (bare CNT fiber) and after coating(coated CNT fiber); and (**B**) electrical resistivity of as-spun and Au doped CNT fiber compared to Au, Cu and Ag wires with similar diameters.

Raman spectra collected on CNT fibers before and after coating reveal the nature of its surface composition. Before coating, typical CNT Raman spectra are observed with G, D and G’ predominant peaks. However, Raman features after polymer coating, shown in Figure 7A, primarily correspond to the predominant peaks of HNBR from literature (2901 cm^−1^ and 1623 cm^−1^) [39].

In order to compare favorably with Cu, a CNT based microcable should have an electrical resistivity of 1.68 × 10^−8^ Ω·m or lower; the typical as-spun CNT fiber has ~5 × 10^−5^ Ω·m. Even after densification, resistivity is still around 1 × 10^−5^ Ω·m, motivating the search for chemical dopants to lower resistivity and improve conductivity (Figure S2). The literature reports that, by exohedral doping, the electrical resistivity of CNT mats and buckypapers has been decreased. However, in most of these cases, resistivity then increases over time; it has been suggested that this increase in resistivity could be the result of the loss of dopant ions or molecules from the CNT surface. Among the more common dopants are HNO_3_, hydrazine, and Au. Particularly in the case of HNO_3_ and hydrazine, dopant concentration appears to decrease with time. As a result of this, the electrical resistivity of the CNT fiber increases to near to their original electrical resistivity. A polymer coating is expected to encapsulate the dopants within the CNT yarns, thereby maintaining the improved electrical resistance, by limiting the release of dopants to the atmosphere. In this work, doping of CNT yarns to increase electrical conductivity was accomplished by using KAuCl_4_ in aqueous solution; electrical resistivity was decreased by up to 83%, as compared to the reference as-spun fiber. The actual resistivity values are in the order of 7 × 10^−6^ Ω·m, determined from hundreds of samples, all of which have shown similar characteristics. After extensive experimentation with Au doping, it has been suggested that the conductivity improvement might be attributed to the Au dopants assisting in the transport of electrons between CNTs.

Electrical characterization of doped CNT fibers, through resistivity measurements prior and subsequent to doping, reveals an order of magnitude reduction in resistivity. Figure 7B shows electrical resistivity of as-spun and doped CNT fibers, as compared to Au, Cu and Ag wires of 25, 25 and 50 μm diameters, respectively. Our home-made setup allows CNT fiber resistivity measurements without causing damage to the cylindrical nature of the CNT fiber. Also, by changing the distance between electrodes in multiples of 2 mm, we can determine the consistency of our resistivity measurements. Most of our measurements were performed on 12 mm long yarns, with the probes contacting in series, except where noted. Voltage is measured across the inner electrodes (4.2 mm apart), while the outer electrodes supply current; outer electrodes are located 2 mm away from the corresponding inner electrode. The CNT fiber is attached to the electrodes using a fast drying colloidal Ag paste.

SEM micrographs from the contact areas reveal some diameter reduction of the CNT fiber near the interface with the electrode. Despite the fast drying nature of the Ag paste, its solvent diffuses within the fiber and causes light densification of the CNTs. Submicron Ag particle accumulations have been observed near the contact points, depending on silver paste droplet size. In case of large droplets, as far as ~200 μm distance of Ag diffusion along the CNT fiber has been observed. This suggests that this diameter variation is attributable to the wetting behavior of the Ag paste on CNT yarn. However, this is observed for as-spun yarns only; densified and doped CNT fibers do not show diameter changes near the contacting electrode. It is concluded that CNT fiber diameter changes will negatively affect the electrical resistivity measurements among samples, but that this affect will be consistent among all of them. Thus, though the absolute resistivity measurement may be incorporate error, the magnitude of the differences between measurements should be accurate.

**Figure 8:**
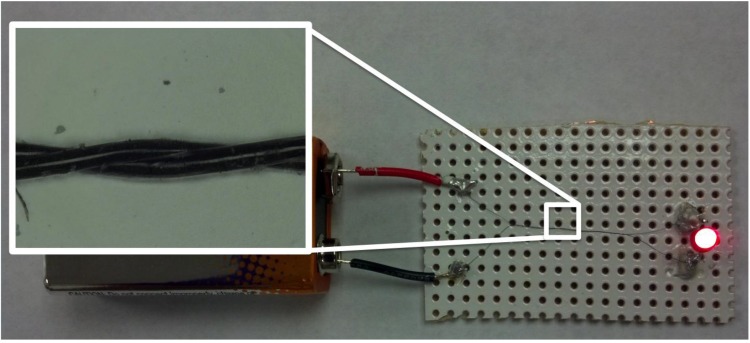
Picture shows a 26.54 µm CNT microcable that is employed to light a battery powered light-emitting diode (LED). The close-up shows two polymer coated CNT fibers that are running side-by-side and they were twisted around each other in a similar manner to normal two pole Cu cable.

To demonstrate the electrical insulation capabilities of our polymer coating, two coated sections were cut and twist around each other in a similar manner to a bipole Cu cable. This section was used to power and LED by connecting to a 9 V battery as shown in Figure 8. The inset image shows the nature of the twisted microcable which is basically made of 26.54 μm individual microcables. With this improved electrical insulation of CNT microcables, we envision several other potential applications for polymer coated CNT fibers. Perhaps the most obvious ones are the applications in coils for electrical motors and transformers. Applications in sensor development and deployment, where micron-size conductors are required to connect with sensors embedded within composite matrixes. Further promising applications are in bio-related implants, where CNT conductivity and biological inertness provide a critical advantage over other implant materials, in addition to the flexibility of small diameter wires and the high resistance to cyclic fatigue.

The coating of CNT yarns may be further improved using surface conditioning processes, such as adding chemical functional groups on the as-spun CNT fiber that would allow the fabrication of microcables with a larger variety of polymers. Chemical functionalization in liquid and plasma functionalization would improve CNT surface wettability; however the creation of C sp^3^ sites on the fiber surface will most likely have a negative effect on the electrical conductivity of the CNT yarns. Different approaches to making contacts between the polymer-coated CNT fibers and metals are being explored. We intend to provide the means to make contacts in a manner similar to the way in which polymer is stripped from electrical cables before making contacts. These studies are currently being performed in the Nanoworld labs, and they will be reported separately.

## 4. Conclusions

We have assembled a thin polymer coating on the CNT fiber surface; this coating is flexible, abrasion resistant, and an excellent insulator. The polymer coating performs the same function as a PVC coating does on common Cu wires. Polymer dip coating is a simple and inexpensive approach to prepare CNT based microcables for use as electrical conductors. A complete coating on the conducting core was achieved, with the qualitative appearance of a uniform coating. Doping attempts on the conducting core caused one order of magnitude reduction of the CNT fiber resistivity, and the coating almost completely eliminated the G band on the Raman spectra. The insulating effectiveness of the polymer coated CNT fibers was demonstrated by the twisting around each other of the two doped and coated CNT fibers in the same manner as a bipolar Cu cable, and using this two-ply microcable to light an LED after connecting to a 9 V battery. We envision that CNT based microcables will find numerous applications, for example, miniature electrical motors and transformers, and may be useful in biological applications where inert thin wires are required.

## Supplementary Materials

Supplementary materials can be accessed at: http://www.mdpi.com/2079-4991/4/4/879/s1.
