# Supplementary material for: Polymer Coating of Carbon Nanotube Fibers for Electric Microcables
Source: Nanomaterials (Basel). 2014 Nov 4;4(4):879–93. doi: 10.3390/nano4040879 (PMC5308460; doi:10.3390/nano4040879)
Supplement: Supplementary file 1 [file nanomaterials-04-00879-s001.pdf]

Supporting Information

Figure S1. Scanning electron microscopy (SEM) image of carbon nanotube (CNT) fiber loops that form as a result of the stress stores within the fiber/thread on the uncoated and polymer coated 2-ply CNT threads.

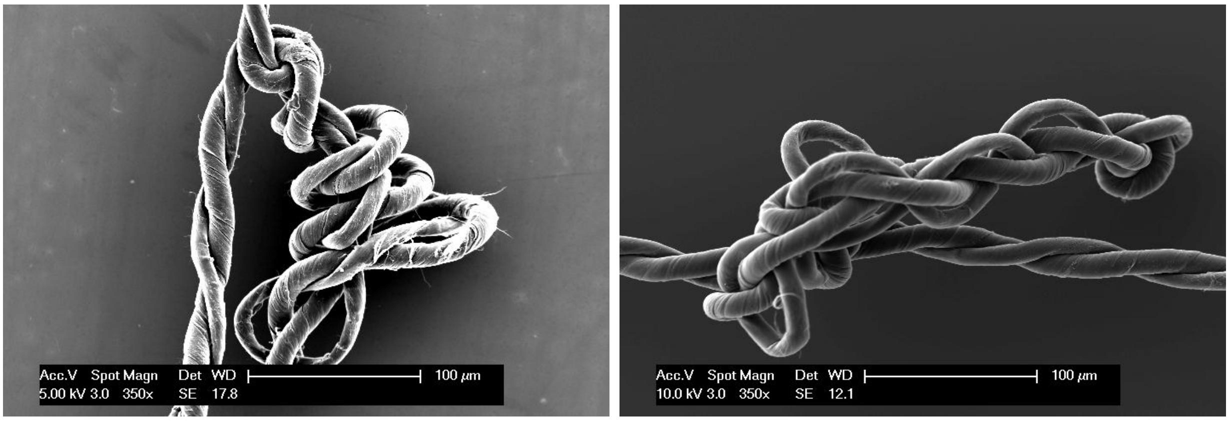

Figure S2. Electrical resistivity measurements of as-spun CNT fibers (reference), acetone densified CNT fibers (solvent densified) and CNT fiber after doping (Au doping).

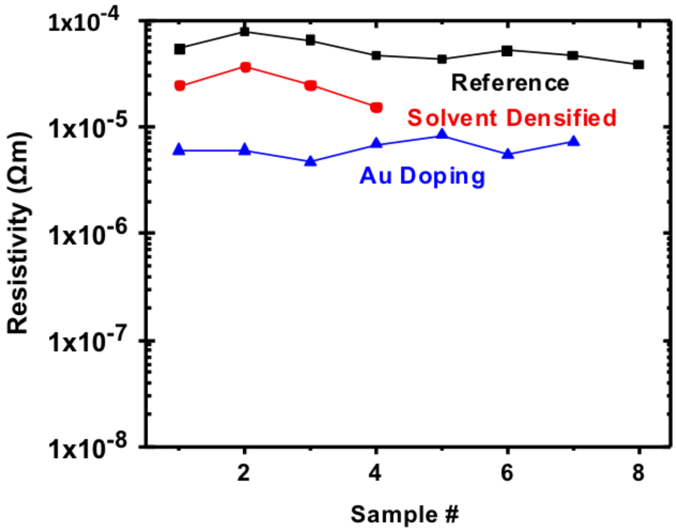

© 2014 by the authors; licensee MDPI, Basel, Switzerland. This article is an open access article distributed under the terms and conditions of the Creative Commons Attribution license (http://creativecommons.org/licenses/by/4.0/).
